# Imaging mass cytometry for high-dimensional tissue profiling in the eye

**DOI:** 10.1186/s12886-021-02099-8

**Published:** 2021-09-20

**Authors:** Anja Schlecht, Stefaniya Boneva, Henrike Salie, Saskia Killmer, Julian Wolf, Rozina Ida Hajdu, Claudia Auw-Haedrich, Hansjürgen Agostini, Thomas Reinhard, Günther Schlunck, Bertram Bengsch, Clemens AK Lange

**Affiliations:** 1 Faculty of Medicine, Eye Center, University of Freiburg, Killianstrasse 5, 79106 Freiburg, Germany; 2 Institute of Anatomy, Wuerzburg University, Wuerzburg, Germany; 3 Faculty of Medicine, Department of Medicine II, Gastroenterology, Hepatology, Endocrinology and Infectious Disease, University Medical Center Freiburg, Freiburg, Germany; 4 Department of Ophthalmology, Semmelweis University, Budapest, Hungary

**Keywords:** Imaging mass cytometry, IMC, multi-dimensional cellular profiling, conjunctival melanoma

## Abstract

**Background:**

Imaging mass cytometry (IMC) combines the principles of flow cytometry and mass spectrometry (MS) with laser scanning spatial resolution and offers unique advantages for the analysis of tissue samples in unprecedented detail. In contrast to conventional immunohistochemistry, which is limited in its application by the number of possible fluorochrome combinations, IMC uses isoptope-coupled antibodies that allow multiplex analysis of up to 40 markers in the same tissue section simultaneously.

**Methods:**

In this report we use IMC to analyze formalin-fixed, paraffin-embedded conjunctival tissue. We performed a 18-biomarkers IMC analysis of conjunctival tissue to determine and summarize the possibilities, relevance and limitations of IMC for deciphering the biology and pathology of ocular diseases.

**Results:**

Without modifying the manufacturer’s protocol, we observed positive and plausible staining for 12 of 18 biomarkers. Subsequent bioinformatical single-cell analysis and phenograph clustering identified 24 different cellular clusters with distinct expression profiles with respect to the markers used.

**Conclusions:**

IMC enables highly multiplexed imaging of ocular samples at subcellular resolution. IMC is an innovative and feasible method, providing new insights into ocular disease pathogenesis that will be valuable for basic research, drug discovery and clinical diagnostics.

**Supplementary Information:**

The online version contains supplementary material available at 10.1186/s12886-021-02099-8.

## Background

For several decades, immunohistochemistry (IHC) has been applied as the gold standard for tissue-specific localization of protein expression for diagnosis of various eye diseases including tumors such as conjunctival melanoma [1]. Existing IHC methods use antibodies tagged with fluorophores or enzyme reporters that generate colored pigments. Using sophisticated equipment, simultaneous staining of up to seven markers for diagnostics is possible [2]. However, due to fluorophore reporter emission overlap, tissue is usually stained with only one to three fluorochrome-tagged antibodies, which is feasible using regular equipment. The number of proteins to be stained is also constrained by the small number of possible donor species of the antibodies. These limitations have so far prevented a multiplex IHC approach in the routine clinical setting.

CyTOF (cytometry by time of flight) analysis was first described in 2009 and combines flow cytometry and mass spectrometry (MS) analysis [3]. This new method uses antibodies or oligonucleotide probes labeled with unique and stable transition element metal isotopes, the signal of which is subsequently amplified by a polymeric metal chelating reagent or metal nanoparticles [3–5]. While this method was previously only applicable for single cell suspensions, the recent combination of mass cytometry with conventional immunohistochemistry (known as Imaging Mass Cytometry, IMC) has led to a next-generation IHC approach that allows the simultaneous staining and analysis of multiple markers [6]. Current mass cytometry instrumentation includes up to 121 different mass detection channels, enabling concomitant multiplex imaging without the risk for overlapping reporter emission [7]. High-resolution scanning laser ablation followed by mass cytometry facilitates highly multiplexed imaging of various tissue types at subcellular resolution [6] using formalin-fixed and paraffin-embedded (FFPE)-material stained with metal-tagged antibodies. This allows for in-depth characterization of diseased tissue to improve diagnostics und treatment options.

In this report, we present a detailed description of the IMC methodology and show the first explorative data on a multiplex characterization approach of ocular tissue at the single cell level. We demonstrate that IMC combined with bioinformatics enables the simultaneous staining and quantification of 18 different proteins in a single tissue section of healthy conjunctival and conjunctival melanoma samples, providing unprecedented insights into disease processes at the cellular level.

## Methods

### Patients

Conjunctival samples were obtained from patients undergoing retinal detachment surgery (n = 1) or conjunctival melanoma resection (n = 1) at the Eye Center of the University of Freiburg. Ethics approval was granted from Ethics Committee of the Albert-Ludwigs-University Freiburg (approval number 481/19).

### Tissue processing

Conjunctival samples (healthy conjunctiva and conjunctival melanoma) were fixed in 4 % formalin for 12 h immediately after surgery and subsequently dehydrated by ascending alcohol series (70 %, 80 %, 2 × 96 % for 30 min, 2 × 100 % for 15 min). After two incubation steps in xylene (one hour each), the samples were incubated in liquid paraffin for 4 h and subsequently embedded. For staining, 6 μm thick sections were made and placed on slides. Prior to staining, paraffin slides were incubated at 60 °C for 90 min and deparaffinized in xylene (2 × 10 min). After rehydration in descending series of ethanol (2 × 100 %, 95 %, 80 % 5 min each) slides were washed in TBS for 5 min. Heat-induced antigen retrieval was performed using DAKO EnvisionFlex target retrieval solution (high pH, Agilent Technologies, Santa Clara, CA) for incubation at 95 °C, 30 min in a pressure cooker. After cooling down for 20 min and washing in TBS, slides were blocked in 3 %BSA in TBS for 60 min at room temperature. The Maxpar® Human Immuno-Oncology IMC™ Panel Kit (Fluidigm, San Francisco, CA) was used to stain the sections. A complete list of antibodies, clones and conjugated metals included in the kit is shown in Table 1. Before staining, we performed a dilution series of all antibodies (1:50, 1:100, 1:200, 1:400, 1:800 and 1:1600) to optimize the protocol. Since the dilution 1:800 gave the most specific staining pattern, we subsequently chose this dilution of antibodies for staining. Diluted antibodies (1:800) were applied to sections simultaneously using an antibody mix and incubated over night at 4 °C in a hydration chamber. After washing in TBS (3 × 5 min), iridium-intercalator solution (1:2000 in TBS) was applied to sections for 5 min and slides were washed 3 × 5 min in TBS afterwards. After 30 min drying at room temperature, sections were subjected to laser ablation and acquisition.

**Table 1:** Antibodies, clones and conjugates

Target	Clone	Metal
CD20	H1	161Dy
CD3	Poly	170Er
CD4	EPR6855	156Gd
CD45Ro	UCJL1	173Yb
CD68	KP1	159 Tb
CD8a	C8/144B	162Dy
FoxP3	236 A/E7	155Gd
Pan-keratin	C11	148Nd
Granzyme B	EPR20129-217	167Er
Ki-67	B56	168Er
PD-1	EPR4877(2)	165Ho
PD-L1	SP142	150Nd
α-SMA	1A4	141Pr
Collagen type I	Poly	169Tm
E-cadherin	24E10	158Gd
Histone H3	D1H2	171Yb
Vimentin	D21H3	143Nd
Nucleic acid		191/Ir/193Ir

### Image acquisition

One image per sample was acquired using the Hyperion Imaging Mass Cytometry™ (IMC™, Fluidigm) after tuning the instrument according to manufacturer’s instructions. Regions of interest were laser-ablated spot-by-spot at 200 Hz resulting in a pixel size/resolution of 1 µm^2^. Preprocessing of the raw data was performed using the CyTOF software v7.0 (Fluidigm). Visualization of images was conducted using MCD Viewer v1.0.560.6 (Fluidigm).

### Segmentation and data analysis

For analysis of multiplex imaging data, such as ion counts per marker, segmentation masks are essential to extract single cell measurements [8]. IMC data was processed as previously described [8]. In brief, text files generated in the process of data acquisition were converted into tiff image stacks using a Python script (https://github.com/BodenmillerGroup/imctools). Hereafter, segmentation masks were developed by using the ilastik software [9] (Version 1.3.2) to designate nuclei, cytoplasm and background fractions. Subsequently the probability maps were uploaded into CellProfiler [10] (Version 3.1.8) to create cell masks which were used to extract single-cell information. The single cell data was normalized to the 99th percentile for Phenograph clustering, which was subsequently performed according to an algorithm implemented in histoCAT[11] (Version 1.76). Data was visualized using histoCAT. t-SNE dimensionality reduction [12] and Phenograph cluster plots [13] were generated.

## Results

### Application of imaging mass cytometry to ocular tissue

**Fig. 1:**
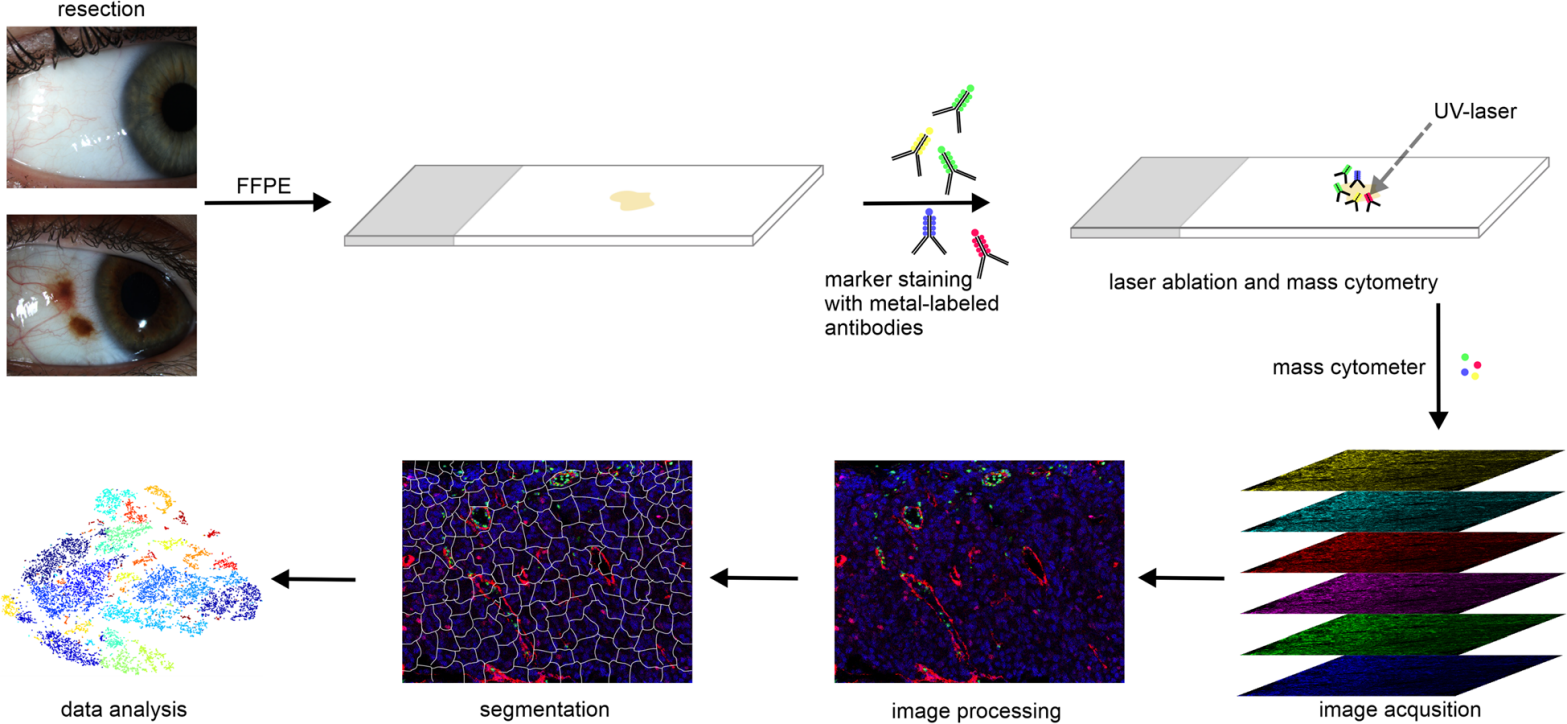
Workflow for imaging mass cytometry on ocular FFPE tissue. Ocular tissue is removed during surgery, followed by fixation, paraffin embedding and sectioning using a microtome. Sections are stained with metal-labeled antibodies. Within the Hyperion Imaging Mass Cytometry system a laser ablates the tissue point by point (spot size 1 µm^2^). Laser ablation and subsequent vaporization creates particle clouds, which are transferred into the mass cytometer by a stream of inert gas. The mass cytometer measures the composition of each particle cloud and reconstructs an image for each stained marker based on the measurement. The resulting images can be processed and analyzed using bioinformatics tools. Cell segmentation allows the analysis of marker expression in each individual cell, providing the data for bioinformatic single-cell analysis

To facilitate the future use of IMC for the examination and diagnosis of ocular tissue, such as conjunctival melanomas, we established a workflow for preclinical and clinical routine, which is shown in Fig. 1. After tissue resection for biopsies or surgery, we immediately fixed the tissue with formalin and embedded it in paraffin and finally cut it into 6 μm thick sections with a microtome. The staining of the sections was performed using the Maxpar® Human Immuno-Oncology IMC™ Panel Kit which was easy to perform within two days according to the manufacturer’s protocol using conventional laboratory equipment. An additional day should be scheduled for measuring the sections with the Hyperion Imaging Mass Cytometry™ system. Thus, this method can be performed in about three days from the time of tissue collection to the generation of the images, which is comparable to the time required for conventional immunohistochemistry, considering the much higher information content due to the simultaneous use of the high number of antibodies on only one tissue slide.

### Evaluation of staining success using Maxpar® Human Immuno-Oncology IMC™ Panel Kit on ocular tissue sections

**Fig. 2:**
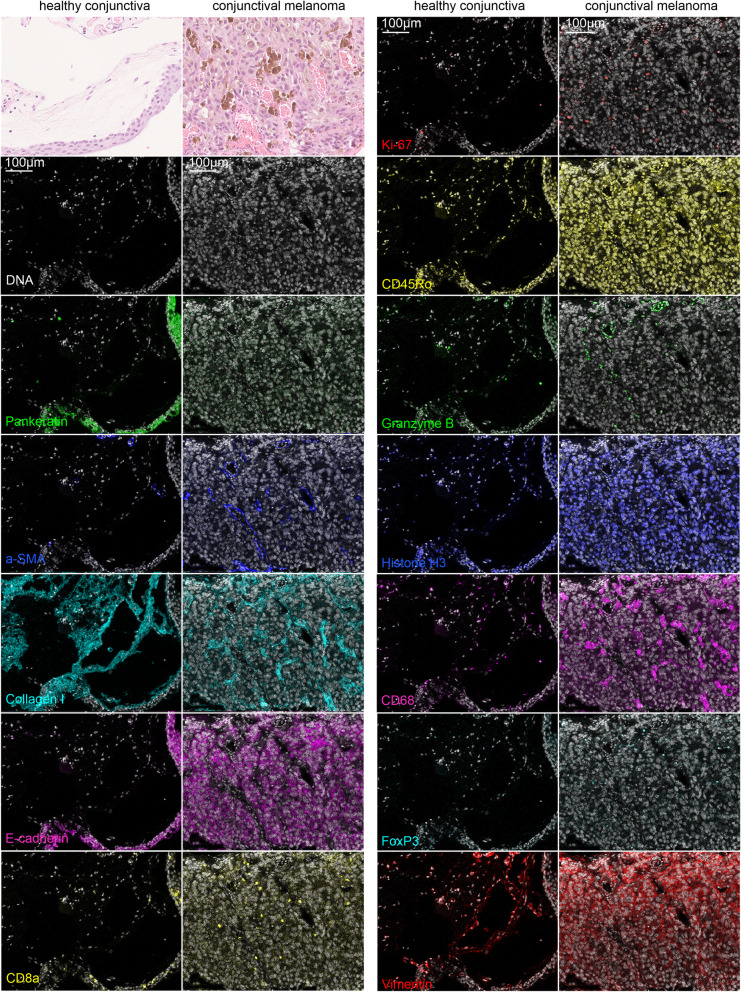
Overview of plausible stainings of healthy conjunctiva and conjunctival melanoma. Nucleic acid labeling (white), respective markers in green, blue, cyan, magenta, yellow, red). Top left: Hematoxylin and eosin (HE) staining of a neighboring section

The Maxpar® Human Immuno-Oncology IMC™ Panel Kit contained 17 markers including structural markers, immune activation and proliferation markers, as well as an intercalator (labeling of nucleic acid) to be used simultaneously. Following the manufacturer’s instructions, we observed positive and distinct staining of 12 / 17 markers (α-SMA, Ki-67, CD8a, CD45Ro, CD68, FoxP3, histone H3, granzyme B, collagen I, E-cadherin, vimentin, pan-keratin, Fig. 2). In contrast, rather diffuse or absent staining of five markers that worked well in control tissues (CD3, CD4, CD20, PD-1, PD-L1, Suppl. Figure 1) was observed in the conjunctiva. The latter is consistent with evidence from the literature showing that PD-1 and PD-L1 expression is very low or absent in conjunctival melanoma tumor cells [14, 15]. However, we cannot be confident in this exploratory study whether the lack of staining is due to the absence of these five markers in the conjunctiva or to technical reasons. Therefore, in the following we only refer to the 12 markers that we were able to stain successfully without major changes to the manufacturer’s protocol.

### Comparison of healthy conjunctiva and conjunctival melanoma

**Fig. 3:**
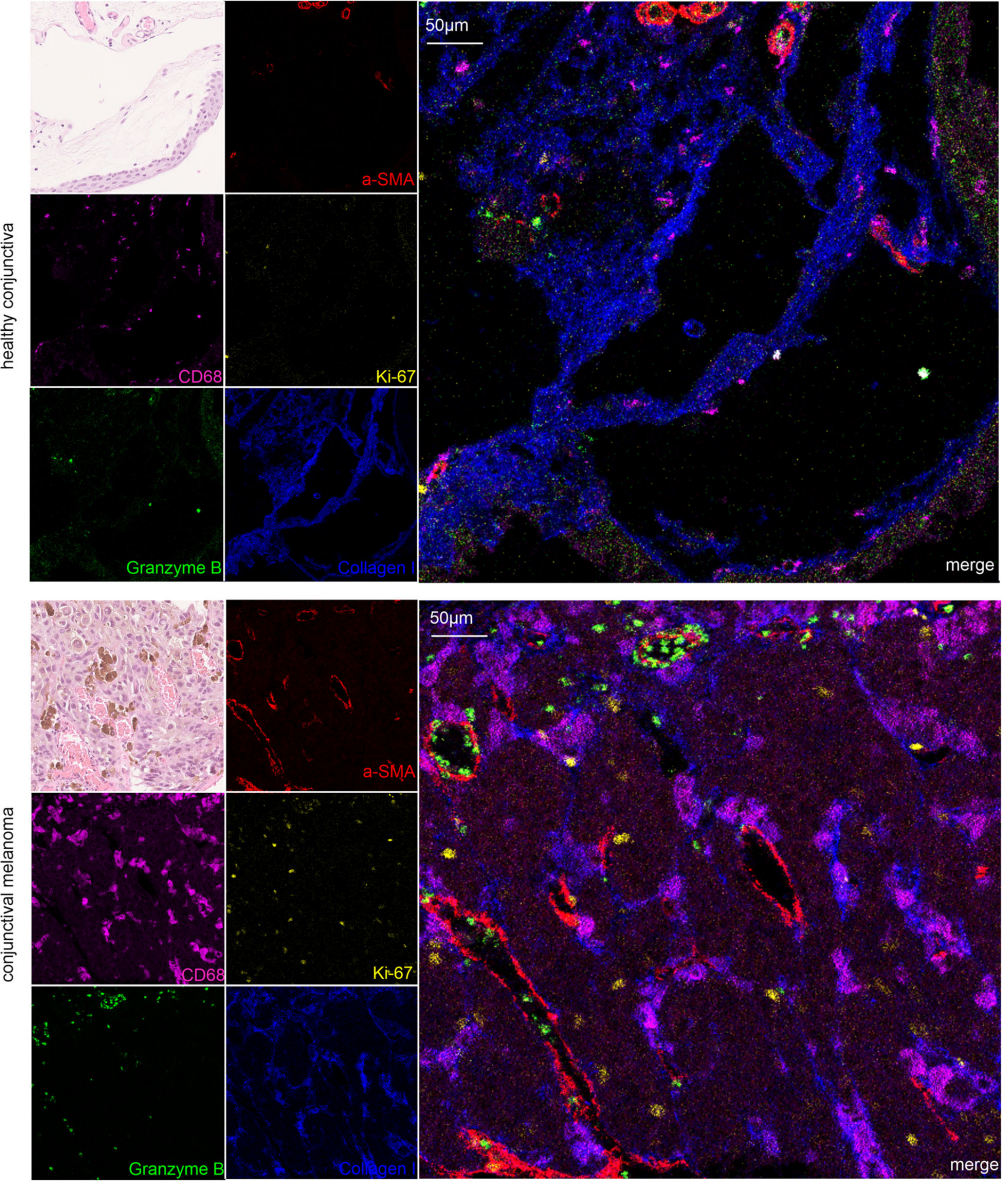
Detection of melanoma-related markers. Immunoreactivity for CD68 (magenta), Ki-67 (yellow) granzyme B (green), α-SMA (smooth muscle actin, red) and collagen I (blue) in the healthy conjunctiva (upper panel) and conjunctival melanoma (lower panel). H & E staining of a neighboring section is shown in the upper left corner

In the next step, we investigated markers in detail that are discussed to play a role in the context of conjunctival melanoma. For this purpose, we analyzed Ki-67 as a proliferation marker [16], CD68 as an myeloid cell marker [17], α-SMA as a marker for perivascular cells, myofibroblasts and smooth muscle cells [18, 19] and granzyme B, which is expressed in cytotoxic T cells [20] in the healthy conjunctiva and in conjunctival melanoma (Fig. 3). The direct comparison of the two tissues regarding these markers revealed considerable differences in immunoreactivity between the two conditions. As expected, hardly any Ki-67, or granzyme B positive cells were found in the healthy conjunctiva, but CD68 positive myeloid cells and α-SMA-positive perivascular smooth muscle cells were readily detected (Fig. 3, upper panel). In contrast, melanoma tissue showed a massive accumulation of CD68-positive immune cells, many proliferating cells and an increased immunoreactivity for granzyme-B and α-SMA. (Fig. 3, lower panel). In summary, analysis of selected markers showed clear differences between the physiological state of the conjunctiva and conjunctival melanoma, which further underlines the validity of this method.

### High-dimensional cellular profiling

**Fig. 4:**
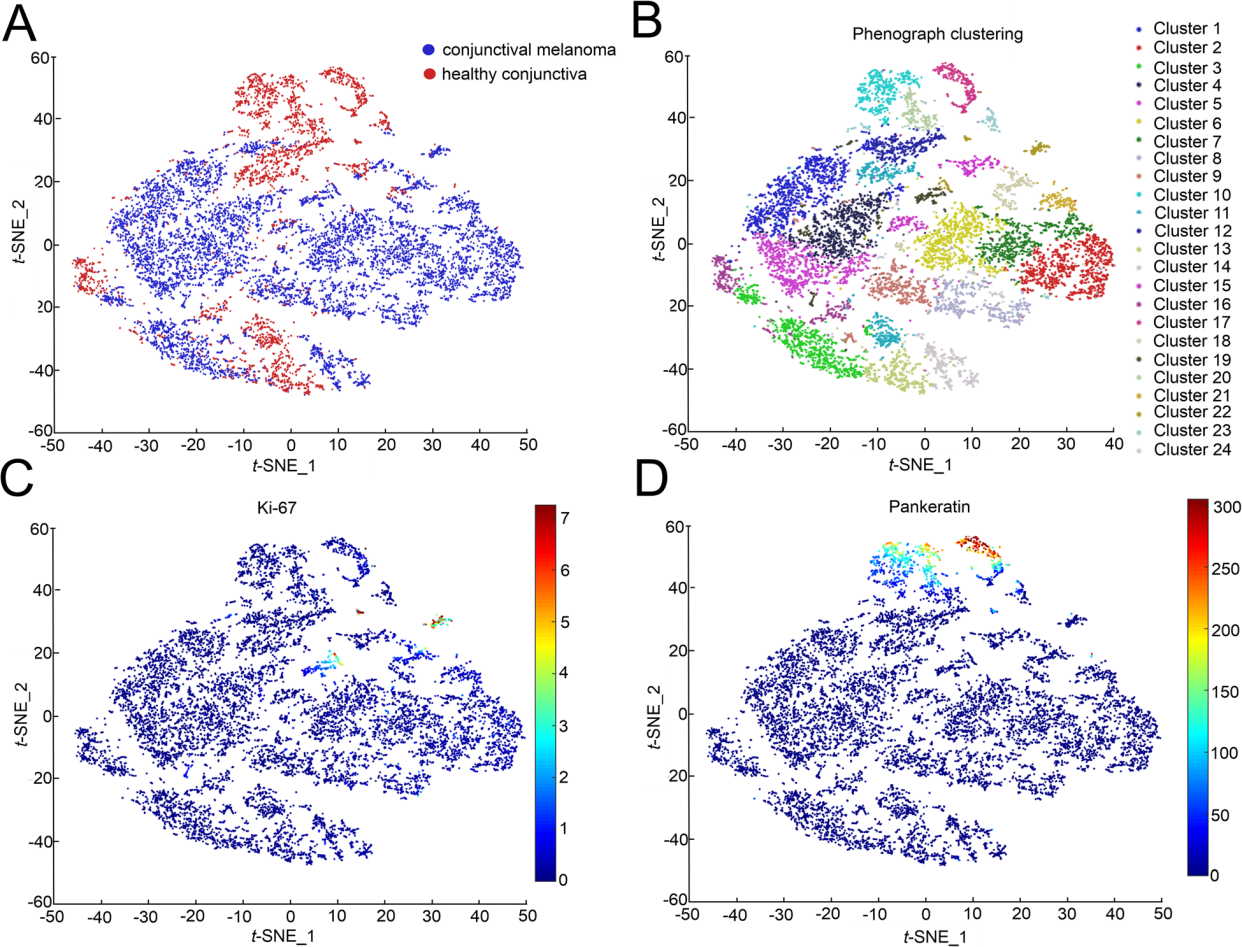
High-dimensional cellular profiling via clustering analysis. Each dot represents a single cell.** A**. t-SNE dimensionality reduction map, representing the different tissue conditions (blue: conjunctival melanoma; red: healthy conjunctiva). **B.** t-SNE map overlaid with the 24 differently colored Phenograph clusters, each color representing one cluster. **C** and **D**. t-SNE plots overlaid with the marker intensities of Ki-67 (**C**) and Pankeratin (**D**) showing Ki-67 and Pankeratin expression in cluster composed almost exclusively of melanoma cells or control conjunctival cells, respectively

To gain a deeper insight into cellular phenotypes and how they differ in healthy conjunctiva compared to conjunctival melanoma, we performed segmentation of images into single cells, which allows the analysis of marker expression in each individual cell, providing high dimensional data for bioinformatic single-cell analysis. The single cell data was normalized to the 99th percentile for Phenograph clustering, which was subsequently performed according to an algorithm implemented in histoCAT [11]. The analysis identified 24 different cellular clusters with distinct expression profiles with respect to the markers used (Fig. 4B). A heatmap illustrating the average marker expression in each cluster is presented in supplemental Fig. 2. Further analysis revealed that some of the clusters can be attributed exclusively to healthy conjunctiva, while other clusters contained predominantly cells from conjunctival melanoma (Fig. 4 A). When focusing on the expression level of distinct factors, we found that, for example, Ki-67 was highly expressed in a cluster composed almost exclusively of melanoma cells (Fig. 4 C). Pankeratin, on the other hand, was mainly expressed in cells derived from healthy conjunctiva (Fig. 4D). These results are consistent with the literature [21–26], clearly demonstrate the validity of IMC, and support the application of the method in ocular research and diagnostics.

## Discussion

Histological visualization of protein expression is an important diagnostic pillar for assessment of various eye diseases [1]. Conventional immunohistochemistry, however, is limited by the small number of available fluorophores, light sources and filters, but also by the scarce number of possible donor species of antibodies, preventing multiplex protein detection. In this study, we introduce Imaging Mass Cytometry (IMC) to the field of ophthalmopathology and show the first explorative data on a multiplex characterization approach of ocular tissue at the single cell level. We demonstrate that IMC combined with bioinformatics enables the simultaneous staining and quantification of 12 different proteins in a single tissue section at the cellular level, providing unprecedented insights into disease processes.

Imaging mass cytometry was first described in 2014 by Bernd Bodenmiller’s group and allows the simultaneous staining of several dozen markers by combining conventional immunohistochemistry with the CyTOF mass cytometer [6]. This technique allows a multidimensional analysis of protein expression in subcellular resolution, which is a great innovation in research as well as in clinical routine diagnostics. A major advantage of this method is that sample preparation is similar to conventional immunohistochemistry, both in terms of sample processing and time investment [6]. Since the technique is based on the detection of metal isotopes and not fluorophore-conjugated antibodies, background autofluorescence is not an issue in this setup and amplification of measured signals is not necessary [6]. A further advantage is the high resolution (1 μm) of the reconstructed images, which is comparable to light microscopic images and surpasses that of other techniques such as MALDI (Matrix Assisted Laser Desorption/Ionization) [27]. The validity of the method is impressive and several studies have shown that conjugation of antibodies with metal isotopes does not affect antigen binding properties and IMC staining patterns could be reproduced by regular IHC [6, 28]. A recent publication even describes a way to visualize and analyze proteins and RNA transcripts simultaneously on a single tissue section using IMC and a metal tag-based in situ protocol [5, 29]. This approach builds a bridge between transcriptomic and proteomic expression, which may massively increase the information content of patient samples and create a new level of tissue analysis [5].

Although IMC has great advantages and has developed remarkably over the last years, it still has some limitations that need to be pointed out. The successful analysis of tissue sections with this method is based, as with conventional IHC, on the specificity of the antibodies used. Firstly, antibodies against the proteins of interest must be available, secondly, a plausible antigen-antibody interaction is a prerequisite for the successful application in IMC [6]. Furthermore, in conventional IHC, the performance of each individual antibody can be optimized by modifying the staining protocol with respect to demasking procedure, incubation time or blocking. In a multiplex approach, where up to 40 antibodies are stained in parallel, different protocols may be unfeasible [6].

Although existing instruments can theoretically measure more than 100 different channels, the multiplex application is currently still restricted to a few dozen due to limited availability of suitable isotopes [3, 6, 30]. Even though IMC allows multidimensional analysis at the single cell level, which is a great innovation for example in tumor research, the analysis of tissue sections is only a snapshot and cannot represent the dynamic processes that occur during tumor development.

It is also important to emphasize that IMC is not an unbiased approach to the analysis of the total proteome, as the information content of the data is clearly limited by the pre-selection of markers to be stained and, furthermore, might be narrowed by measurement speed. Since the data acquisition takes two hours per square millimeter of tissue, analysis of smaller regions is often preferred due to time constraints [31]. As tumor tissues are frequently characterized by high heterogeneity, the analysis might be biased by a reduced representativeness [30]. Not only the measurement, but also the analysis of the generated data requires a considerable amount of time and financial effort due to its multidimensionality. Therefore, a routine diagnostic application of IMC in daily practice may currently still be challenging to implement.

Even if IMC at present still has some limitations, such as the extensive data acquisition time, the number of isotopes available or the complex bioinformatic analysis, it can be assumed that this will change within the next few years. Lower costs and innovative developments, which increase availability of isotopes may lead to a situation where in the future more than 100 markers can be measured in less than one hour [6].

## Conclusions

In summary, Imaging Mass Cytometry represents a highly multiplexed imaging method which allows the analysis of small ocular tissue samples with subcellular resolution in so far unpreceded detail. Thus, IMC can be considered as the next generation immunohistochemistry, which enables the investigation of molecular changes in neoplastic and degenerative eye diseases and may even play a role in clinical diagnostics in the future.

## Supplementary information

Additional file 1 **Supplemental Fig. 1: **Overview of markers showing diffuse or absent staining in healthy conjunctiva and/or conjunctival melanoma. Nucleic acid labeling is shown in blue, respective markers in white. 

Additional file 2 **Supplemental Fig. 2:** Heatmap illustrating average marker expression in each cluster.

## Data Availability

The datasets used and/or analyzed during the current study is available in the zenodo respiratory, 10.5281/zenodo.5136291.

## Abbreviations

CyTOF: Cytometry by time of flight
FFPE: Formalin-fixed and paraffin-embedded
IHC: Immunohistochemistry
IMC: Imaging mass cytometry
MS: Mass cytometry
