# Supplementary figures and images for: Imaging mass cytometry for high-dimensional tissue profiling in the eye
Source: BMC Ophthalmol. 2021 Sep 20;21:338. doi: 10.1186/s12886-021-02099-8 (PMC8454101; doi:10.1186/s12886-021-02099-8)

healthy conjunctiva

conjunctival melanoma

DNA only

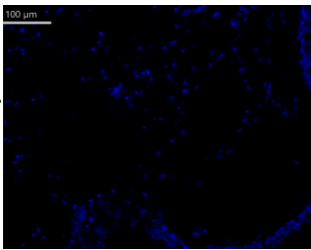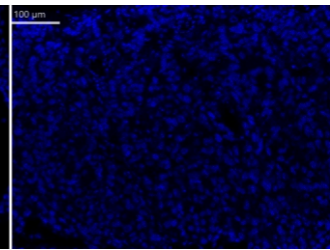

CD3

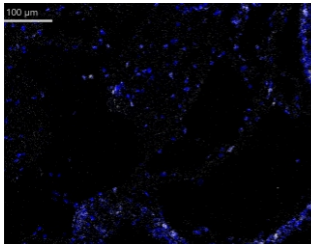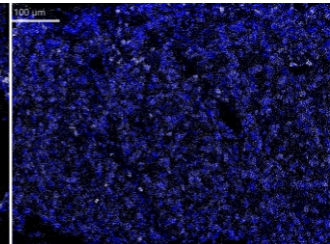

CD4

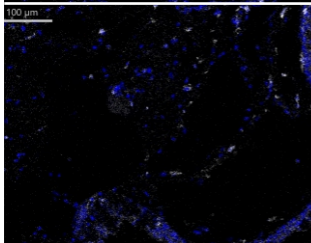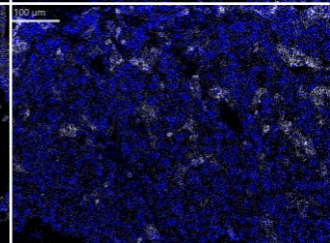

CD20

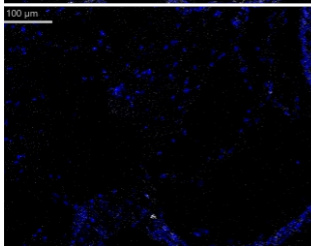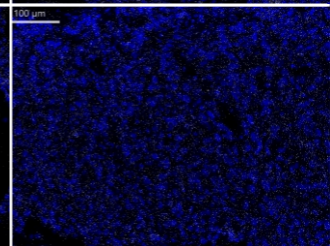

PD-1

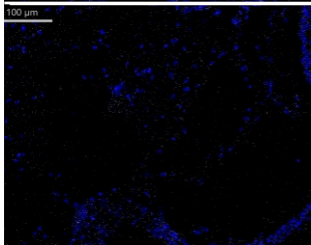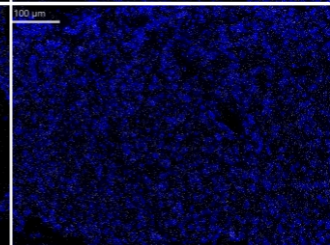

PD-L1

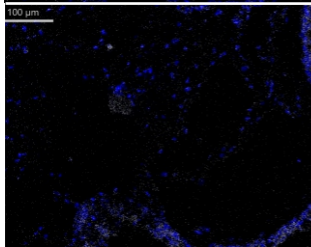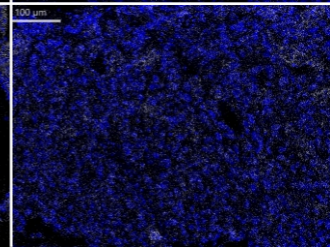

Supplement: Supplementary file 1 — Supplemental Fig. 1: Overview of markers showing diffuse or absent staining in healthy conjunctiva and/or conjunctival melanoma. Nucleic acid labeling is shown in blue, respective markers in white. [file 12886_2021_2099_MOESM1_ESM.pdf]

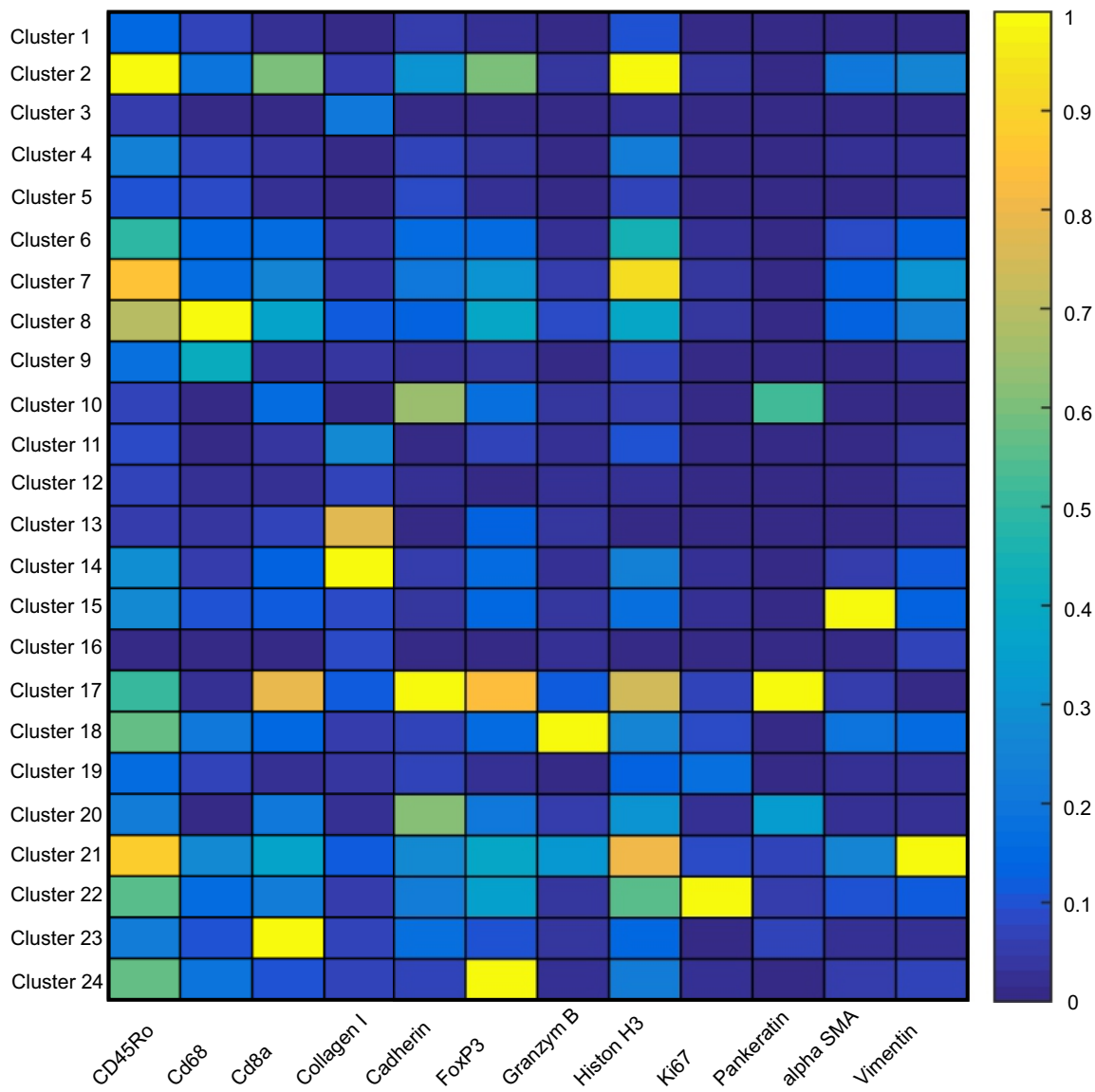

Supplement: Supplementary file 2 — Supplemental Fig. 2: Heatmap illustrating average marker expression in each cluster. [file 12886_2021_2099_MOESM2_ESM.pdf]
